# The impact of sleep disorders on microvascular complications in patients with type 2 diabetes (SLEEP T2D): the protocol of a cohort study and feasibility randomised control trial

**DOI:** 10.1186/s40814-021-00817-z

**Published:** 2021-03-22

**Authors:** Christina Antza, Ryan Ottridge, Smitaa Patel, Gemma Slinn, Sarah Tearne, Matthew Nicholls, Brendan Cooper, Asad Ali, Abd A. Tahrani

**Affiliations:** 1 Institute of Metabolism and Systems Research, University of Birmingham, Birmingham, UK; 2 Birmingham Clinical Trials Unit, College of Medical and Dental Sciences, University of Birmingham, Birmingham, UK; 3 Lung Function & Sleep, University Hospitals Birmingham NHS Foundation Trust, Birmingham, UK; 4 Department of Respiratory Medicine, University Hospitals Coventry & Warwickshire, Coventry, UK; 5 Centre of Endocrinology Diabetes and Metabolism, Birmingham Health Partners, Birmingham, UK; 6 Department of Diabetes and Endocrinology, University Hospitals NHS Foundation Trust, Birmingham, UK

**Keywords:** Obstructive sleep apnoea, Type 2 diabetes mellitus, Continuous positive airway pressure, Randomised control trial, Chronic kidney disease, Nephropathy, Neuropathy, Retinopathy, Sight-threatening retinopathy, Diabetic foot

## Abstract

**Background:**

Obstructive sleep apnoea (OSA) is very common in patients with type 2 diabetes (T2D). We and others have shown that OSA was associated with diabetes-related microvascular complications in patients with T2D in cross-sectional and longitudinal studies and that compliance with continuous positive airway pressure (CPAP) reduced the progression of microvascular complications. Hence, we hypothesised that adequate CPAP reduces the development of microvascular complication in patients with T2D.

**Methods:**

SLEEP T2D is a cohort study with embedded feasibility, open-label, parallel-arm, randomised control trial (RCT) over 2 years. The primary aim is the feasibility of conducting a definitive RCT assessing the impact of CPAP on chronic kidney disease and other microvascular complications in patients with T2D. The main parameters are to assess willingness of participants to be randomised, follow-up rates, CPAP adherence/compliance, to optimise the choice of outcome measures for a substantive trial, and to identify the parameters for sample size calculations. The secondary aims of the study are related to the impact of CPAP, sleep-related disorders, and sleep chronotype on a variety of diabetes-related end points. The study participants were recruited from the T2D services in multiple NHS trusts across England. The main exclusion criteria for the cohort study are as follows: T1D, eGFR < 15 mL/min/1.73 m^2^, known OSA, active malignancy or chronic kidney disease from reasons other than diabetes, pregnancy, professional drivers, and a history of falling asleep whilst driving within last 2 years. The main exclusion criteria from the RCT were as follows: Apnoea-Hypopnoea Index < 10 and Epworth Sleepiness Score ≥ 11. Study participants were extensively phenotyped clinically and biochemically. The OSA diagnosis was based on multichannel portable device (ApneaLink Air^TM^, Resmed).

**Discussion:**

The feasibility RCT will help us design the future RCT to assess the impact of CPAP on diabetes-related microvascular complications. The cohort study will generate preliminary data regarding the impact of sleep quality, duration, and chronotype on diabetes-related outcomes which could lead to further mechanistic and interventional studies.

**Trial registration:**

ISRCTN, ISRCTN12361838. Registered 04 April 2018, Protocol version: v5.0 02.12.19.

**Supplementary Information:**

The online version contains supplementary material available at 10.1186/s40814-021-00817-z.

## Background

### Obstructive sleep apnoea

Obstructive sleep apnoea (OSA) is a chronic disorder characterised by complete or partial collapse of the upper airway during the sleep, which results in recurrent cyclical changes in oxygen saturations (de- and re-saturations), heart rate, blood pressure, and intrathoracic pressure [1]. In addition, OSA is characterised by hypoxemia, fragmented sleep, and increased sympathetic nervous activity [2, 3].

Earlier estimates of OSA prevalence were 2–4%, but with increasing prevalence of obesity, OSA prevalence has also increased [4, 5]. The Wisconsin sleep study showed that 24% of men and 9% of women had OSA (defined as an Apnoea-Hypopnoea Index (AHI) > 5 events/h) and 9% of men and 4% of women had moderate to severe OSA (AHI ≥15 events/h) [6]. More recently, the prevalence of OSA (AHI ≥ 15 events/h) was estimated to be 17% and 9% in men and women aged 50–70 years [7].

### Obstructive sleep apnoea and type 2 diabetes mellitus

OSA is an established independent risk factor for incident type 2 diabetes (T2D) [8–10], and more recently T2D has been shown to be associated with increased risk of OSA compared to appropriately matched population without diabetes and after adjustments for potential confounders [11]. Hence, unsurprisingly, multiple studies showed a high prevalence of OSA in patients with T2D (23–86%) [12–17] with prevalence being higher in White Europeans compared to South Asians [18]. Our previous work demonstrated an OSA prevalence of 65% in patients with T2D [15]. In addition, the severity of OSA is independently associated with worsening glycaemic control in patients with T2D [19].

### Diabetes-related microvascular complications

Diabetes-related microvascular complications (foot, kidney, eyes, and neuropathy) have significant impact on patients with T2D and are associated with increased morbidity and mortality [20]. In addition, most of the financial burden of diabetes in the NHS is due to its complications (the cost of treating diabetes complications is expected to be £13.5 billion by 2035/36) [21]. As a result, there is significant interest in reducing microvascular complications to reduce the health and economic burden of diabetes. Therefore, there is an interest in better understanding the pathogenesis of diabetes-related complications in order to develop new treatment strategies and reduce the burden of diabetes on patients, society, and the NHS.

Hyperglycaemia induced oxidative and nitrosative stress are essential steps in the pathogenesis of microvascular complications resulting in DNA damage, excessive poly (ADP-ribose) polymerase (PARP) activation, and inhibition of glyceraldehyde 3-phosphate dehydrogenase resulting in activation of aldose reductase, protein kinase C (PKC), the hexosamine pathway, and advanced glycation end products (AGE), all of which result in endothelial and microvascular dysfunction resulting in microvascular complications [22–24]. Data from people without diabetes has shown that OSA can result in similar molecular consequences to those of hyperglycaemia including increased oxidative and nitrosative stress, activation of PKC and AGE production, increased inflammation, and endothelial dysfunction [25–45]. Our previous work has expanded those findings to patients with T2D; OSA and nocturnal hypoxemia were associated with nitrosative stress, oxidative stress, PARP activation, and impaired microvascular regulation in patients with T2D and OSA compared to those with T2D only [15, 46]. Therefore, it is plausible that OSA might aggravate or contribute to the development of microvascular complications.

### Obstructive sleep apnoea and microvascular complications in type 2 diabetes

Cross-sectional studies showed that diabetes-related chronic kidney disease (CKD), peripheral neuropathy (PN) and sight-threatening retinopathy (STR) were more common in patients with OSA and T2D compared to those with T2D alone despite adjustment for a wide range of variables [15, 46, 47].

After a follow-up of 2.5 years, the decline in estimated glomerular filtration rate (eGFR) (based on the 4-variable MDRD equation) was greater in patients with T2D and OSA compared to patients with T2D only [46, 47]. Baseline OSA and AHI were independent predictors of study-end eGFR after adjustment for baseline eGFR, age, obesity, diabetes duration, medications, and other confounders [46].

OSA was also an independent predictor of progression to pre-proliferative/proliferative STR after adjustment over a 5-year follow-up period [47]. More recently, our team found in a cohort matched controlled population-based study from the UK that the risk of developing cardiovascular disease, foot disease, diabetic PN, CKD, and death in patients with T2D and OSA was greater than those with T2D but without OSA (data undergoing peer review).

Observational data showed that 3 months’ treatment with continuous positive airway pressure (CPAP) statistically significantly increased the eGFR and decreased the serum creatinine levels in patients with OSA [48]. Our observational data also showed that patients with T2D and OSA who were compliant with CPAP had less development of proliferative retinopathy over 5 years and less decline in eGFR over 2.5 years compared to those non-compliant with CPAP or with mild OSA, despite lack of significant differences in participants’ characteristics between those compliant and non-compliant with CPAP [46, 47].

Taken together, these data suggest that OSA could potentially be a modifiable risk factor for the development and/or worsening of microvascular complications in patients with T2D. Hence, randomised controlled trials (RCTs) will be needed to further explore these findings.

## Methods/design

**Fig. 1:**
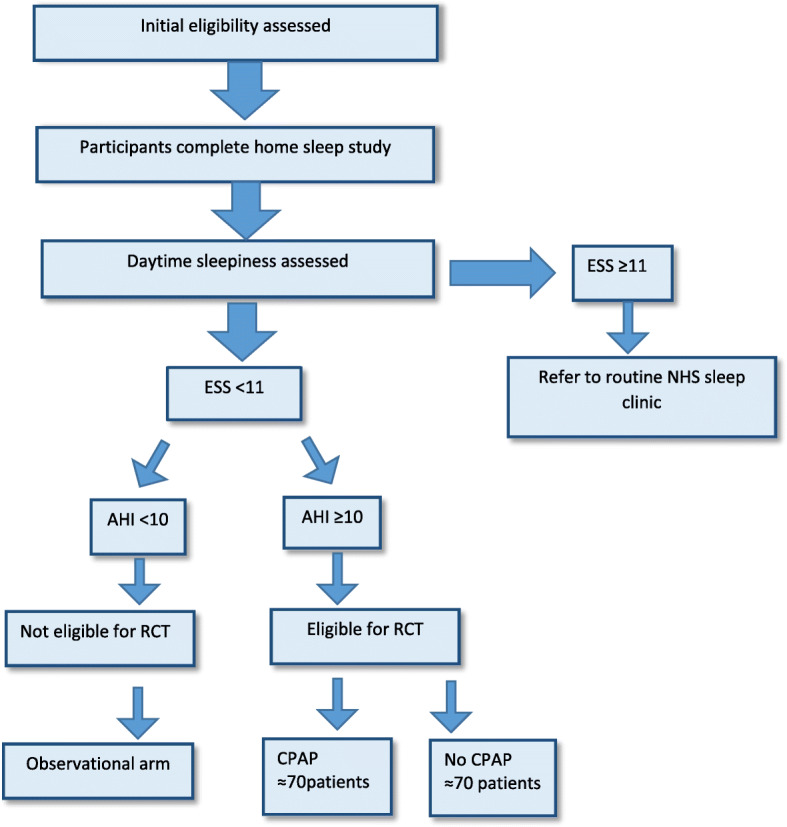
Participants pathway through the study. ESS, Epworth Sleepiness Scale; AHI, Apnoea-Hypopnoea Index; RCT, randomised control trial, CPAP, continuous positive airway pressure

SLEEP T2D is an observational cohort study with embedded feasibility RCT. Participants are recruited to the RCT from the baseline population of the cohort study. The overall study design is summarised in Fig. 1. The RCT is an open-label, randomised controlled, parallel arm, clinical trial of patients with T2D and OSA. Participants will be randomised in a 1:1 ratio to either CPAP or no CPAP for 2 years. The observational cohort study will also run over up to 2 years and will include all patients who consented to the study regardless of OSA status or randomisation status. Patients will be recruited to the RCT based on OSA being diagnosed by the baseline sleep study. Patients who are not randomised and are in the cohort will continue to be in the cohort and will not be randomised even if they develop OSA during the follow-up.

All participants are recruited and followed up at their local OP clinic. Participants’ routine care is not adjusted in this study. All participants will receive their routine health care in their local centres for the whole duration of the study. Scoring of the sleep studies and CPAP initiation and follow-up were performed by a sleep physiologist (MN) and was supported by a senior sleep physiologist (BC) and a sleep consultant physician (AA) when needed.

### The study rationale

#### The RCT rationale

The primary aim of this study (SLEEP T2D) is to assess the feasibility of running a definitive RCT in patients with T2D and OSA, randomising participants to CPAP vs. no CPAP. The overall aim of the substantive RCT would be to determine the impact of OSA treatment (CPAP) on the progression of CKD and microvascular complications in patients with T2D.

The reason for not conducting a substantive RCT straight away is that we want to ensure that we can meet the pre-defined criteria (listed below) or understand what may need to be amended before proceeding to a full RCT; in particular, we want to specifically examine acceptability of randomisation to patients, the ability to obtain enough follow-up data, and the ability to achieve high CPAP compliance. Poor compliance with CPAP is very common in real life as well as in research settings [49], and this could undermine the outcome of the future RCT, hence we want to examine whether we can achieve high compliance with CPAP (see details below) and learn of what may be needed to achieve our targets.

Patients with moderate to severe OSA are recommended to be treated with CPAP in the UK if they are symptomatic, particularly if they have excessive daytime sleepiness (EDS) [50]. Therefore, randomising patients with EDS to no CPAP would raise ethical concerns. To avoid this, the Epworth Sleepiness Scale (ESS) will be used to assess the presence of EDS. Patients with EDS (ESS ≥ 11) will be excluded from the RCT and their general practitioner and/or diabetologist will be informed as these patients are likely to require treatment. Patients who have OSA without EDS will be included in the RCT if they meet the RCT inclusion/exclusion criteria (please see below). Excluding patients with ESS ≥ 11 is more stringent than other CPAP RCTs in the field of OSA in order to maintain patient safety [51–53].

An AHI of ≥ 10 was chosen as the cut off for randomisation as we felt patients will not tolerate CPAP with lower values of AHI and because some other CPAP trials used the same cut off for randomisation [54–56].

The control arm is ‘no CPAP’. The choice of no CPAP over sham CPAP was a pragmatic one. Using sham CPAP is more expensive and more time and resource consuming. It is difficult to blind patients or the assessors to the treatment allocation as patients on sham CPAP will still have symptoms related to OSA. This lack of effect can be associated with treatment non-compliance, which will also affect outcomes. Furthermore, although sham CPAP produces much less pressure than active CPAP, this pressure is not 0 and hence might still have some physiological function [57, 58]. The use of no CPAP rather than sham CPAP is common in CPAP trials [57–59].

The study duration of 2 years is because many of the secondary outcomes such as renal function or neuropathy do not change significantly over short period of times.

#### The rationale for the cohort study

Sleep T2D will collect a large amount of data on study participants whether they are randomised or not. Hence, in addition to the RCT, Sleep T2D will include a cohort study assessing the impact of sleep-related parameters on diabetes-related outcomes (see outcomes below). Whilst multiple studies have shown the relationship between sleep quality, duration, and chronotype with haemoglobin A1c (HbA1c) [60–65] and one recent study eGFR [66], there are no published cohort studies at the time of writing the protocol that assessed the impact of sleep quality, duration, and chronotype and EDS on glycaemic control and other diabetes-related outcomes in people with T2D. We hypothesised that short sleep duration, poor sleep quality, and later chronotype are associated with worse diabetes-related outcomes. Sleep T2D will examine these potential associations longitudinally. In addition, the data will allow us to explore the interactions between different sleep parameters/disorders and T2D-related outcomes. The findings of the cohort study might lead to future RCTs and all the outcomes of the cohort study are either secondary or tertiary (see below).

### Study participants

Patients with T2D will be recruited from the outpatient diabetes departments of 13 NHS trusts in England.

### Inclusion and exclusion criteria

The following are the inclusion criteria for the observational cohort study:

1. Are ≥ 18 years old,
2. Diagnosis of T2D,
3. eGFR ≥ 15 mL/min/1.73 m^2^ in last 12 months.

The following are the exclusion criteria for the observational cohort study:

1. History of T1D,
2. Known OSA, active malignancy or CKD from reasons other than diabetes,
3. Receiving chemotherapy, immunosuppressant drugs, or home oxygen treatment,
4. History of recurrent hospital admissions due to infective exacerbation of a respiratory condition,
5. Received contrast imaging within the last 2 months,
6. Pregnancy,
7. Intending to undergo bariatric surgery during the study duration,
8. Unable to comply with the study protocol,
9. Unable to give informed consent,
10. Professional drivers, operators of heavy machinery, and/or working at high altitude,
11. History of falling asleep whilst driving within last two years.

Participants will be considered eligible for randomisation if they:

1. Are willing to be randomised to CPAP or no CPAP,
2. Have an ESS < 11 (as completed by the participant during the baseline assessment),
3. Have an AHI ≥ 10*.

Potential participants will be excluded from the RCT if they have:

1. A resting oxygen saturation < 90%*, or
2. Central apnoea index > 15/h*
  *as detected during the baseline home-based sleep study using multichannel portable device.

When in doubt regarding eligibility for randomisation and/or the sleep recordings, the cases will be discussed with the study sleep physician (AA) and senior sleep physiologist (BC) to ensure patient safety is not compromised.

### Randomisation

Eligible participants for the RCT will be randomised in a 1:1 ratio to either CPAP or no CPAP. A stratified block randomisation method will be used within the computerised randomisation system to ensure balance in the treatment allocation over the following variables: ethnicity (White Europeans, others), gender (male, female), and severity of OSA (AHI 10– < 15, ≥ 15).

### RCT Treatment arms

Intervention arm: Participants that are allocated to the CPAP arm will be provided with a CPAP machine (ResMed Airsense 10 Autoset™). Each patient in the CPAP arm will be given an appropriate mask, connecting hose, a heated humidifier, and all the necessary accessories to operate the CPAP equipment for the duration of the trial. This machine features built-in wireless connectivity capability to monitor compliance remotely via a secured website, AirView (AV); AutoRamp™ with sleep onset detection; expiratory pressure relief and Easy-Breathe technology, allowing a suitably qualified person to remotely change settings to improve patient compliance and comfort if required.

Control arm: Νο treatment.

### Outcome measures

The primary outcome measures are to:

1. Assess willingness of participants to be randomised,
2. Assess willingness of clinicians to recruit participants,
3. Assess follow-up rates and adherence/compliance rates,
4. Provide data to inform the sample size for a substantive trial,
5. Optimise the choice of outcome measures for a substantive trial.

The secondary outcome measures are:

To assess the impact of CPAP in patients with T2D on:

1. Measures of CKD including eGFR, cystatin-C, and albumin/creatinine ratio (ACR),
2. Diabetes-related neuropathy (including painless and painful peripheral neuropathy, cardiac autonomic neuropathy, and peripheral autonomic neuropathy),
3. Diabetes-related retinopathy and maculopathy,
4. Metabolic parameters such as weight, HbA1c, blood pressure, and lipids profile.

The changes in the above-listed diabetes-related endpoints will be described as changes in the values of continuous variables or changes in categories for categorical variables.

The tertiary outcome measures are:

1. To explore the utility of different biomarkers in screening for OSA in patients with T2D,
2. To explore the mechanisms via which CPAP might have an impact on diabetes-related complications,
3. To assess the relationship between other sleep-related disorders, metabolic parameters, and vascular disease in patients with T2D,
4. To build up a cohort of well characterised patients for longitudinal follow-up to inform us about the natural history of OSA, sleep-related disorders, and diabetes-related complications.

### Feasibility criteria

1. Recruiting the proposed sample size within the planned time frames,
2. Meeting the proposed time frames in regard to interpreting the sleep assessments and initiating patients on treatment (within 8 weeks from the registration),
3. Achieving a follow-up rate ≥ 80% for randomised patients,
4. Achieving a CPAP usage ≥ 4 h/night on ≥ 70% of nights in ≥ 80% patients randomised to CPAP treatment,
5. Generating a mean and standard deviation regarding the predicted response to the intervention to allow sample size calculations for a substantive RCT.

### Compliance initiation and monitoring

CPAP will be initiated by the sleep physiologist (MN) at a place that is convenient to the patients (at home for example). Prior to initiating CPAP, the physiologist will discuss the benefits of CPAP treatment and will provide explanation about the technical aspects and usage of the device. MN will contact the patients by telephone 2–3 times during the first week of CPAP use to troubleshoot any challenges.

Adequate compliance in this trial will be defined as an average usage of CPAP > 4 h/night on 70% of nights. Compliance will be monitored using the manufacturer’s AirView remote monitoring, whilst only the study sleep technician and chief investigator will have permission to remotely access and change CPAP settings via the secured website using AirView. The sleep technician and chief investigator will view sleep data of all patients remotely twice weekly on average and will identify those using CPAP < 4 h. Patients will be called if CPAP usage is < 4 h per night to determine the cause and any action required to enhance compliance.

### Study assessments and follow-up

Following consent and checking eligibility, participants will have a baseline assessment (0 months), in which the following data will be collected:

Physical examination:

- Height and weight measurements,
- Waist, neck and hip circumferences,
- Blood pressure.

Biochemistry:

- HbA1c,
- Random lipid profile,
- Creatinine,
- Urinary ACR.
- A serum sample for the measurement of cystatin C will also be taken.

Quality of life:

- Short Form Health Survey (SF-12) questionnaire.

Peripheral neuropathy:

- Michigan Neuropathy Screening Instrument (MNSI) questionnaire,
- Short Form McGill Pain Questionnaire (SF-MPQ),
- Selected sites will also perform Sudoscan and Cardiac autonomic neuropathy (CAN) assessments.

Sleep apnoea, habits, duration, and quality:

- One-night home-based sleep assessment using a portable multi-channel respiratory device approved for screening and diagnosing OSA (ApneaLink Air, ResMed),
- ESS,
- Berlin questionnaire,
- Morningness-Eveningness Questionnaire (MEQ),
- Pittsburgh Sleep Quality Index (PSQI).

Retinopathy

The evaluation of retinopathy will be assessed using 2 × 45 degrees’ digital retinal images per eye as per the English National Screening program guidelines [67].

The examinations and the questionnaires for the baseline assessment are described in the Supplementary File 1 in detail.

All patients in the RCT and cohort study will be contacted every 6 months by phone to enhance follow-up rates and to collect data about any new relevant developments and ensure continuation of consent and patient safety. Those in CPAP will be contacted more often as described above depending on compliance and also for the short term after CPAP initiation. The same data with the baseline visit will be collected at study-end (at up to 2 years from baseline). Table 1 summarises the procedure from registration to follow-up visits.

**Table 1:** Outcome measures and assessments

Summary of assessments	Trial period			
	Baseline visit (0)	Allocation [1] RCT only	Follow-up (telephone)	Follow-up visit (2)
Time point	0	1 m ± 1 m	6 m, 12 m, 18 m ± 2 m	2 years ± 3 m
**Registration and treatment allocation**				
**Main eligibility assessed**	x			
**Study consent**	x			
**Registration onto study**	x			
**Additional eligibility for RCT assessed**		x		
**Consent RCT**		x		
**Treatment allocation/randomisation**		x		
**GP letter(s)**	x	x		
**Intervention** (if receiving)				
**CPAP Initiation (CPAP use continues until 2 years)**		x		
**Assessments**				
**QoL Questionnaire** SF12	x			x
**General patient information:** Patient details	x			
Review of medication	x			x
Review of past medical history	x			
**Physical exam and blood pressure:** Height, weight , blood pressure (standardised)	x			x
Circumferences hip, waist, neck (standardised)	x			x
**Routine biochemistry:** lipid profile and HBA1c, eGFR and creatinine, plasma urea, electrolytes and creatinine, ACR	x^a^			x^a^
**Additional blood for** future biomarker studies; **Additional blood for** serum cystatin C	x			x
**Peripheral neuropathy**: MNSI; SF-MPQ; vibration perception threshold; monofilament and Neuropad test	x			x
**SUDOSCAN and CAN** assessment	x selected sites			x selected sites
**Retinopathy**	x			x
**Sleepiness and obstructive sleep apnoea risk:** One night home-based sleep assessment	x			x
ESS and Berlin Questionnaire	x			x
**Sleeping habits, duration and quality:** MEQ and PSQI	x			x
**6 monthly follow-up form**			x	
**SAE monitoring** (SAE forms)	x	x	x	x

*RCT* randomised control trial, *CPAP* continuous positive air pressure, *SF-12* Short Form Health Survey, *HBA1c* haemoglobin A1c, *eGFR* estimated glomerular filtration rate, *ACR* albumin/creatinine ratio, *CAN* cardiac autonomic neuropathy, *ESS* Epworth Sleepiness Scale, *PSQI* Pittsburgh Sleep Quality Index, *SAE* serious adverse event

^a^If performed within 3 months of visit (within 12 months for ACR) then previously recorded measurements can be used

### Statistical considerations and rational

Since this is a feasibility RCT, no formal sample size calculations have been undertaken as the study is not designed or powered to detect a statistically significant difference in efficacy between the two treatment arms, which is consistent with guidance from the National Institute for Health Research (NIHR) in the UK [68].

As the cohort study is independent of the RCT’s aim, it is intended to be hypothesis generating, and no formal sample size calculation has been undertaken for it. One hundred forty patients will be randomised in the RCT. At least 60% of patients attending a secondary care diabetes clinic could be eligible for recruitment and based on the results of our previous work, showing an OSA prevalence of 65% in patients with T2D [15], it is expected that up to 500 patients will need to be registered into the observational cohort study and assessed for OSA to reach 140 participants with confirmed OSA eligible for randomisation in the RCT.

### Statistical analysis

The primary comparison groups will be composed of those treated with CPAP versus those not receiving CPAP. In the first instance, all analyses will use the intention to treat principle.

Analyses of outcomes will primarily take the form of simple descriptive statistics (e.g. proportions and percentages, means and standard deviations) and, where appropriate, point estimates of effect sizes (e.g. mean differences and relative risks) and associated 95% confidence intervals.

For the tertiary objectives, the full cohort will be analysed.

### Monitoring

#### Safety reporting (RCTs only)

The collection and reporting of adverse events (AEs) will be in accordance with the UK Policy Framework for Health and Social Care Research and the requirements of the Health Research Authority. The investigator will assess the seriousness and causality (relatedness) of all AEs experienced by the trial participant. Any trial-related serious AEs will be reported by the investigator or delegate on a trial-specific serious AE form to the Birmingham Clinical Trial Unit trials office, no later than 24 h after first becoming aware of the event.

#### Independent committee

A joint study oversight committee that comprises both Study Steering Committee and Data Monitoring Committee functions will be engaged for this trial. The role of the study oversight committee is to provide the overall supervision of the trial, monitor trial progress and advice on scientific credibility.

### Ethics approval and consent to participate

Patients will be approached by their clinical care teams and then consent for the cohort study will be obtained and baseline assessments performed by appropriately trained research nurses as per the delegation log. If eligible for the RCT, a further informed consent will be obtained by post or in person and the randomisation visit can be performed either at their local research centre or by telephone.

The study will be performed in accordance with the recommendations guiding physicians in biomedical research involving human subjects, adopted by the 18th World Medical Association (WMA) General Assembly, Helsinki, Finland, 1964, amended by the 48th WMA General Assembly, Somerset West, Republic of South Africa, 1996.

The trial will be conducted in accordance with the Research Governance Framework for Health and Social Care, the applicable UK Statutory Instruments (which include the Data Protection Act 2018 and Human Tissue Act 2008), the EU General Data Protection Regulation 2018, and the principles of Good Clinical Practice. Written informed consent will be provided by all patients prior to any trial-related procedures. Participants will be free to withdraw from the trial at any time without any effect on their standard of care.

## Discussion and conclusions

SLEEP T2D is an observational cohort study with a subset of participants included in a feasibility RCT of a CE marked medical device used within its intended purpose. The outcome of this study will guide the design of a future RCT that might have significant impact on reducing the burden of T2D microvascular complications. In addition, the observational cohort study will provide novel hypothesis generating data that might lead to future RCTs to improve diabetes-related outcomes.

This study has several strengths including its novelty and the multicentre and randomised design. It also uses a pragmatic approach to CPAP treatment and the study population are very well characterised and phenotyped. However, the study has its limitations, including the unblinded design, but most of the study outcomes are not based on patient reporting and the decision not to have sham CPAP was pragmatic and discussed above. The study has now finished recruitment and all patients are currently in follow-up. As a result of the disruption caused by COVID-19 pandemic and the focus on COVID-19 research by the participating NHS trusts and the need to move the feasibility RCT forward to apply for funding for the full RCT (if applicable), we have submitted an amendment that has been approved to collect study-end data as soon as it is practical and allowed by the NHS trusts. This means that for some study participants, the study-end visit will be after less than 2 years follow-up and for others it will be more than 2 years. We aim to start the study end visits from January 2021 in the centres that allow us. We expect all patients to have at least 1 year follow-up by the time they have the study end visit.

## Supplementary Information

**Additional file 1.** Detailed description of the outcome measures and assessments.

## Data Availability

Not applicable.

## Abbreviations

OSA: Obstructive sleep apnoea
AHI: Apnoea-Hypopnoea Index
T2D: Type 2 diabetes mellitus
PARP: Poly (ADP-ribose) polymerase
PKC: Protein kinase C
AGE: Advanced glycation end products
CKD: Chronic kidney disease
PN: Peripheral neuropathy
STR: Sight-threatening retinopathy
eGFR: Estimated glomerular filtration rate
CPAP: Continuous positive airway pressure
RCT: Randomised control trial
EDS: Excessive daytime sleepiness
ESS: Epworth Sleepiness Scale
HbA1c: Haemoglobin A1c
ACR: Albumin/creatinine ratio
SF-12: Short Form Health Survey
MNSI: Michigan Neuropathy Screening Instrument
SF-MPQ: Short Form McGill Pain Questionnaire
CAN: Cardiac autonomic neuropathy
MEQ: Morningness-Eveningness Questionnaire
PSQI: Pittsburgh Sleep Quality Index
AE: Adverse event
