# Supplementary material for: The impact of sleep disorders on microvascular complications in patients with type 2 diabetes (SLEEP T2D): the protocol of a cohort study and feasibility randomised control trial
Source: Pilot Feasibility Stud. 2021 Mar 22;7:80. doi: 10.1186/s40814-021-00817-z (PMC7982768; doi:10.1186/s40814-021-00817-z)
Supplement: Supplementary file 1 — Additional file 1. Detailed description of the outcome measures and assessments. [file 40814_2021_817_MOESM1_ESM.docx]

**Supplement File 1.** Detailed description of the outcome measures and assessments.

1) The physical examination will include:

-Circumference measurements

Waist, hip and neck circumferences will be measured using an inelastic measuring tape. All circumferences will be taken with the subjects standing upright, with the face directed straight, and shoulders relaxed. Two measurements will be entered into the CRF (for average calculation). Waist circumference will be measured at the midpoint between the inferior border of the ribcage and the superior aspect of the iliac crest. Hip circumference will be measured horizontally at the widest circumference of the hips. Neck circumference will be measured in the midway of the neck, between mid-cervical spine and mid-anterior neck. In men with a laryngeal prominence (Adam's apple), it will be measured just below the prominence.

-Height and weight measurements

Height will be measured to the nearest 0.1 cm with a rigid stadiometer. Body weight will be measured in light indoor clothing to the nearest 0.1 kg

-Blood Pressure

BP will be measured by an automated device with the patient in sitting position and the arm resting on a table. Two measurements will be taken at least 20 minutes apart with the first measurement to be taken about 20 minutes after the start of the consultation. The two readings will be entered into the CRF (for average calculation).

2) The biochemistry examination will include:

-Haemoglobin A1c (HbA1c) and lipids (routine care, using retrospective data if available)

HbA1C (including percentage, if locally calculated) and total cholesterol, triglycerides, high density lipoprotein (HDL) and low density lipoprotein (LDL) (where LDL is locally available) will be recorded.

-Estimated **G**lomerular **F**iltration **R**ate (eGFR) (routine care, using retrospective data if available)

Serum creatinine levels will be recorded. The eGFR will be calculated by the study database from the creatinine value supplied using the MDRD equation (175 x creatinine^-1.154^ x age^-0.203^ x 0.742 [if female] x 1.212 [if black], creatinine measured in mg/dL). Serum creatinine measurements should be avoided during acute illness or following imaging that used contrast. Note that the eGFR value used to check patient eligibility can be taken from patient notes within 12 months of baseline; however, the serum creatinine level recorded at baseline should be taken from within 3 months of the visit. eGFR will be categorised as the standard stages of chronic kidney disease (REF) ([1](#_ENREF_1)).

-Urine Albumin (routine care, using retrospective data if available)

Albuminuria will be assessed using a single measurement of urine albumin. Microalbuminuria will be defined as albumin-to-creatinine ratio (ACR ACR >3.4mg/mmol and macroalbuminuria as ≥30mg/mmol ([2](#_ENREF_2), [3](#_ENREF_3)). If the patient has a urinary tract infection then ACR will be measured when free from infection. Please note that an ACR value is not required before registration.

For routine biochemistry (HbA1c, lipids, creatinine, and urinary ACR), recent results (within 3 months from baseline, or 12 months for ACR) can be used from patient notes; otherwise fresh samples will be collected. These will require 1 yellow top tube (for lipids and creatinine, approximate volume 8mL) and/or 1 purple top (for HbA1c; approximate volume 8mL), and/or a morning urine sample (approximate volume 20 mL) for urinary ACR. These tests will be performed by the local NHS laboratory as per routine care.

3) Additional samples for biomarker analysis will be taken at sites with the capacity to do so and include:

-Serum Cystatin C

A serum sample for the measurement of cystatin C will be taken to analyse for early markers of diabetic CKD, for storage at designated research laboratories at University of Birmingham.

-Additional biomarker blood samples

Additional blood samples will be taken for storage at designated research laboratories at University of Birmingham for analysis for obstructive sleep apnea (OSA) biomarkers and potential mechanisms of the impact of OSA on the study outcomes.

4) Quality of Life

A general health related quality of Life measure will be used, the Short Form Health Survey (SF-12).

5) Peripheral Neuropathy will be assessed through:

-The Michigan Neuropathy Screening Instrument (MNSI)

MNSI is a validated questionnaire and foot examination that has been used in several landmark epidemiological studies ([4-7](#_ENREF_4)). The questionnaire component (MNSIq) comprises 15 questions ([5](#_ENREF_5)). The examination component (MNSIe) comprises a limited foot inspection to identify deformity, skin abnormalities, and ulceration, coupled with an assessment vibratory perception at the great toe (measured using a 128 Hz tuning fork) and ankle tendon reflexes ([5](#_ENREF_5)). Diabetes-related peripheral neuropathy will be diagnosed if the MNSI examination score was > than 2 and/or MNSI questionnaire score was ≥ 7 ([8](#_ENREF_8)).

-Short Form McGill Pain Questionnaire (SF-MPQ)

The SF-MPQ is used to assess the presence and severity of painful neuropathy ([9](#_ENREF_9)).

-Neuropad (routine care)

The indicator test (Neuropad) is a plaster which is applied to the sole of the feet just below the 1^st^ and 2^nd^ toes of both feet. The Neuropad is a non-invasive method to assess sudomotor function. While the MNSI mainly assess large fibre function, the Neuropad is closely related to small fibre function. The Neuropad is based on the colour change of a cobalt II compound from blue to pink after 10 minutes exposure to dermal foot perspiration at the plantar foot regions ([10](#_ENREF_10)). The change in colour will be recorded in the database as none, partial or complete and the time to the start of colour change will also be recorded. A complete change in colour is considered normal ([8](#_ENREF_8)).

-Vibration perception

Vibration perception will be tested using a biothesiometer on the great toe of each foot; the average of three measurements will be taken ([11](#_ENREF_11)).

-10-g monofilament test

The perception to a 10-g monofilament (applied to 10 positions, the tip of each toe, under 3 metatarsal heads, the plantar surface of the foot and the dorsal space between the first and second toe) will be used as a test for foot insensitivity; an abnormal monofilament test is defined as <8 correct responses ([12](#_ENREF_12)).

6) Additional Peripheral Neuropathy at Selected Sites:

SUDOSCAN

SUDOSCAN assesses sudomotor function through galvanic skin response. This test is similar to Neuropad in that it measures sweat production in a non-invasive way. It provides an accurate evaluation of sweat gland function. Patients place their hands and feet on stainless-steel sensor plates, and an incremental low direct voltage (lower than 4V) is applied for two to three minutes. Measurement is based on an electrochemical reaction between the sensor plates and chlorides of the sweat after being stimulated by the low-level voltage. Quantitative results are expressed as Electrochemical Skin Conductances (ESC, in microsiemens, µS) for the hands and feet. Training will be provided by the company and the chief investigator. Due to the limitations of equipment availability the SUDOSCAN test will only be performed at selected sites. SUDOSCAN results will be interpreted as per the study by VInik et al that established normative values ([13](#_ENREF_13)) and will also be examined as continuous variables

Patients with pacemakers, defibrillators or similar implantable devices, who have had a whole hand or foot amputated, or who have an active foot ulcer will be excluded from the SUDOSCAN test

-Cardiac Autonomic Neuropathy

Cardiac autonomic neuropathy (CAN) will be assessed using heart rate variability and will be analysed using the continuous wavelet transform methods to generate numerical and graphical data using the ANX 3.0 software, ANSAR Inc., Philadelphia, USA. Details can be found in our previous work ([14](#_ENREF_14)). Heart rate variability and blood pressure are recorded with the patient in sitting position during resting, deep breathing, Valsalva manoeuvre and standing position ([15](#_ENREF_15)). A diagnosis of CAN will be made when 2 or more of the following tests are abnormal: E/I ratio, Valsalva ratio, 30:15 ratio and postural drop in blood pressure (drop of 20mmHg in systolic or 10mmHg in diastolic blood pressure) ([16](#_ENREF_16)). Age-related normal values were defined as previously reported ([17](#_ENREF_17)).

The test will be performed while the patient is in sitting position and in the fasting state if possible. If not fasting, caffeine intake should be avoided for 2 hours prior to the test. Due to the limitations of equipment availability the CAN test will only be performed at selected sites.

Training on how to perform the CAN will be provided by the chief investigator. The raw data will be analysed on the CAN laptop and a summary sheet of results will be printed off and for purposes of this trial will be classed as source data.

Patients with pacemakers, defibrillators or similar implantable devices will be excluded from doing the CAN test.

7) Sleep and Obstructive Sleep Apnea will be assessed through:

-One night home-based sleep assessment

Presence and severity of OSA will be assessed by performing a one night home-based sleep assessment using a portable multi-channel respiratory device approved for screening sleep apnoea (ApneaLink Air, ResMed). Portable devices are used widely in sleep apnoea research including my previous work ([18-20](#_ENREF_18)). The device records oral/nasal airflow, chest movements, oxygen saturations, and heart rate.

The ApneaLink Air device comes with Airvew diagnostics. This is supplied by ResMed and is similar to AV (see Section 7) but is linked with the sleep assessment and not continuous positive air pressure (CPAP). In Airvew diagnostics the study centres will download the sleep assessment from the device once returned by patient. Once downloaded, the sleep assessment will become accessible via a remote website. The sleep studies will be scored and interpreted by the centrally based Sleep Technician remotely; where there are any queries raised by the Sleep Technician (e.g. uncertainty about how to score a particular respiratory event) Dr Asad Ali, a consultant in sleep medicine and/or Prof. Brendan Cooper, both part of the study team, will be consulted and a consensus reached. They will only be provided with anonymised data; they will, where required, review data from all sites.

The Sleep Technician will print off the report, classed as source data, and return to the SLEEP T2D Trials Office. The principal investigator will be informed of the results of the sleep assessment with recommendations by the chief investigator or delegate.

The sleep assessment will give, amongst other data, the Apnea- Hypopnea Index (AHI), the average number of apnoea and hypopnoea events per hour, which is routinely used to classify the severity of OSA to mild, moderate, and severe (5-14.9, 15-29.9 and ≥30 respectively).

NOTE: Patients receiving CPAP will not require a sleep assessment at their final visit as the CPAP machine records the data required.

-The Epworth Sleepiness Scale (ESS)

ESS, an 8 item questionnaire, is a measure of day time sleepiness ([21](#_ENREF_21)). ESS correlates with sleep latencies ([21](#_ENREF_21)) and with measures of OSA severity ([22](#_ENREF_22)).

-The Berlin questionnaire

The Berlin Questionnaire focuses on a limited set of known risk factors and symptoms for OSA; one introductory question and four follow-up questions concern snoring; three questions address daytime sleepiness, with a sub-question about sleepiness behind the wheel and one question concerns history of high blood pressure and obesity (based on BMI) ([23](#_ENREF_23)).

-Sleeping Habits, Duration and Quality

Data regarding sleep habits, duration and quality will be collected using the Horne Ostberg Morningness-Eveningness questionnaire (MEQ) ([24](#_ENREF_24)) and the Pittsburgh Sleep Quality Index (PSQI) ([25](#_ENREF_25)).

8) Retinopathy

Diabetic retinopathy (DR), maculopathy and sight threatening DR are assessed using 2 x 45 degrees digital retinal images per eye as per the English National Screening program guidelines ([26](#_ENREF_26)). This should be retrospective data take as part of routine care.

The image grades will be obtained from the patient’s electronic records, from the national diabetic retinopathy screening program, direct from the appropriate screening centre, or from the letter sent to the patient informing them of their grades. The latest grade prior to each visit will be recorded.

Most clinical centres should have access to the retinopathy grades via one of the methods above and will be able to check this directly. If required, the patients will be asked to bring in a letter of their grade to the centre at each visit. In cases where the patient letter is used, a copy of the letter will be taken and inserted in the patient’s medical notes.

Abbreviations: **HbA1c:** Haemoglobin A1c, **HDL:** High Density Lipoprotein, **LDL:** Low Density Lipoprotein, **eGFR:** Estimated Glomerular Filtration Rate, **ACR:** Albumin-to-Creatinine Ratio, **OSA:** Obstructive Sleep Apnoea, **SF-12:** Short Form Health Survey, **MNSI:** Michigan Neuropathy Screening Instrument, **SF-MPQ:** Short Form McGill Pain Questionnaire, **CAN:** Cardiac Autonomic Neuropathy, **CPAP:** Continuous Positive Air Pressure, **AHI:** Apnoea- Hypopnoea Index, **MEQ:** Morningness-Eveningness questionnaire, **DR:** Diabetic Retinopathy

References

1. The Renal Association. CKD Stages. [Available from: <https://renal.org/health-professionals/information-resources/uk-eckd-guide/ckd-stages>.

2. Parving HH, Lewis JB, Ravid M, Remuzzi G, Hunsicker LG, investigators D. Prevalence and risk factors for microalbuminuria in a referred cohort of type II diabetic patients: a global perspective. Kidney Int. 2006;69(11):2057-63.

3. Pugliese G, Solini A, Fondelli C, Trevisan R, Vedovato M, Nicolucci A, et al. Reproducibility of albuminuria in type 2 diabetic subjects. Findings from the Renal Insufficiency And Cardiovascular Events (RIACE) study. Nephrol Dial Transplant. 2011;26(12):3950-4.

4. Boyraz O, Saracoglu M. The effect of obesity on the assessment of diabetic peripheral neuropathy: a comparison of Michigan patient version test and Michigan physical assessment. Diabetes Res Clin Pract. 2010;90(3):256-60.

5. Feldman EL, Stevens MJ, Thomas PK, Brown MB, Canal N, Greene DA. A practical two-step quantitative clinical and electrophysiological assessment for the diagnosis and staging of diabetic neuropathy. Diabetes care. 1994;17(11):1281-9.

6. Martin CL, Albers J, Herman WH, Cleary P, Waberski B, Greene DA, et al. Neuropathy among the Diabetes Control and Complications Trial cohort 8 years after trial completion. Diabetes care. 2006;29(2):340-4.

7. Factors in development of diabetic neuropathy. Baseline analysis of neuropathy in feasibility phase of Diabetes Control and Complications Trial (DCCT). The DCCT Research Group. Diabetes. 1988;37(4):476-81.

8. Tahrani AA, Ali A, Raymond NT, Begum S, Dubb K, Mughal S, et al. Obstructive Sleep Apnea and Diabetic Neuropathy. American Journal of Respiratory and Critical Care Medicine. 2012;186(5):434-41.

9. Melzack R. The short-form McGill Pain Questionnaire. Pain. 1987;30(2):191-7.

10. Kempler P, Amarenco G, Freeman R, Frontoni S, Horowitz M, Stevens M, et al. Management strategies for gastrointestinal, erectile, bladder, and sudomotor dysfunction in patients with diabetes. Diabetes Metab Res Rev. 2011;27(7):665-77.

11. Elliott J, Tesfaye S, Chaturvedi N, Gandhi RA, Stevens LK, Emery C, et al. Large-fiber dysfunction in diabetic peripheral neuropathy is predicted by cardiovascular risk factors. Diabetes care. 2009;32(10):1896-900.

12. Pambianco G, Costacou T, Strotmeyer E, Orchard TJ. The assessment of clinical distal symmetric polyneuropathy in type 1 diabetes: a comparison of methodologies from the Pittsburgh Epidemiology of Diabetes Complications Cohort. Diabetes Res Clin Pract. 2011;92(2):280-7.

13. Vinik AI, Smith AG, Singleton JR, Callaghan B, Freedman BI, Tuomilehto J, et al. Normative Values for Electrochemical Skin Conductances and Impact of Ethnicity on Quantitative Assessment of Sudomotor Function. Diabetes technology & therapeutics. 2016;18(6):391-8.

14. Piya MK, Shivu GN, Tahrani A, Dubb K, Abozguia K, Phan TT, et al. Abnormal left ventricular torsion and cardiac autonomic dysfunction in subjects with type 1 diabetes mellitus. Metabolism. 2011;60(8):1115-21.

15. Colombo J, Shoemaker WC, Belzberg H, Hatzakis G, Fathizadeh P, Demetriades D. Noninvasive monitoring of the autonomic nervous system and hemodynamics of patients with blunt and penetrating trauma. J Trauma. 2008;65(6):1364-73.

16. Vinik AI, Ziegler D. Diabetic cardiovascular autonomic neuropathy. Circulation. 2007;115(3):387-97.

17. Ziegler D, Laux G, Dannehl K, Spuler M, Muhlen H, Mayer P, et al. Assessment of cardiovascular autonomic function: age-related normal ranges and reproducibility of spectral analysis, vector analysis, and standard tests of heart rate variation and blood pressure responses. Diabet Med. 1992;9(2):166-75.

18. Punjabi NM, Caffo BS, Goodwin JL, Gottlieb DJ, Newman AB, O'Connor GT, et al. Sleep-disordered breathing and mortality: a prospective cohort study. PLoS Med. 2009;6(8):e1000132.

19. Tahrani AA, Ali A, Raymond NT, Begum S, Dubb K, Altaf QA, et al. Obstructive sleep apnea and diabetic nephropathy: a cohort study. Diabetes care. 2013;36(11):3718-25.

20. Tahrani AA, Zeng W, Shakher J, Piya MK, Hughes S, Dubb K, et al. Cutaneous structural and biochemical correlates of foot complications in high-risk diabetes. Diabetes care. 2012;35(9):1913-8.

21. Johns MW. A new method for measuring daytime sleepiness: the Epworth sleepiness scale. Sleep. 1991;14(6):540-5.

22. Johns MW. Reliability and factor analysis of the Epworth Sleepiness Scale. Sleep. 1992;15(4):376-81.

23. Netzer NC, Stoohs RA, Netzer CM, Clark K, Strohl KP. Using the Berlin Questionnaire to identify patients at risk for the sleep apnea syndrome. Ann Intern Med. 1999;131(7):485-91.

24. Horne JA, Ostberg O. A self-assessment questionnaire to determine morningness-eveningness in human circadian rhythms. Int J Chronobiol. 1976;4(2):97-110.

25. Buysse DJ, Reynolds CF, 3rd, Monk TH, Berman SR, Kupfer DJ. The Pittsburgh Sleep Quality Index: a new instrument for psychiatric practice and research. Psychiatry Res. 1989;28(2):193-213.

26. Harding S, Greenwood R, Aldington S, Gibson J, Owens D, Taylor R, et al. Grading and disease management in national screening for diabetic retinopathy in England and Wales. Diabet Med. 2003;20(12):965-71.
